# Evaluation of Assisted Reproductive Technology Outcomes Using Crossover Administration of Two Recombinant Follicle-Stimulating Hormones in the Same Patients

**DOI:** 10.7759/cureus.85312

**Published:** 2025-06-03

**Authors:** Shoji Kokeguchi, Eri Okamoto, Yuri Mizusawa, Hiroaki Shibahara, Kazuki Yamagami, Kohyu Furuhashi, Masahide Shiotani

**Affiliations:** 1 Medical Physics, Hanabusa Women's Central Fertility Clinic, Kobe, JPN; 2 Laboratory, Hanabusa Women's Central Fertility Clinic, Kobe, JPN

**Keywords:** crossover, follitropin alfa, follitropin delta, ovarian stimulation, recombinant fsh

## Abstract

Objective: This crossover study compared the effects of two recombinant follicle-stimulating hormone (rFSH) preparations, follitropin alfa and follitropin delta, on assisted reproductive technology (ART) outcomes.

Methods: Patients aged 25-42 years (body mass index: 18-30, anti-Müllerian hormone level ≤2.03 ng/mL) underwent two oocyte retrieval cycles, one with follitropin alfa and the other with follitropin delta, and were divided into four groups based on the crossover use of these preparations. Eighty-one patients received follitropin alfa during both cycles. Fifty-one patients received follitropin delta during both cycles. Forty-four patients received follitropin delta during the first cycle and follitropin alfa during the second cycle. Thirty patients received follitropin alfa during the first cycle and follitropin delta during the second cycle.

Results: There was no significant difference in the rate of increase in the number of oocytes retrieved between the first and second cycles, regardless of whether follitropin alpha or follitropin delta was used initially. No significant difference in the blastocyst formation rate was observed. However, cycles in which follitropin alfa was used for the first stimulation and follitropin delta for the second showed a significantly smaller decline in oocyte yield compared to cycles that began with follitropin delta followed by follitropin alfa.

Conclusion: The present study concluded that in non-polycystic ovary syndrome (PCOS) patients with an anti-Müllerian hormone (AMH) level of ≤2.03 ng/mL - classified as intermediate responders - the use of follitropin delta (12 μg) in the second in vitro fertilization (IVF) cycle resulted in a significantly smaller change in the number of retrieved oocytes compared to the first cycle. This suggests that the use of follitropin delta in the second cycle may be a viable and proactive option in such cases.

## Introduction

Recently, the approach to controlled ovarian stimulation (COS) has shifted from emphasizing maximum stimulation and maximum oocyte yield to prioritizing individualized stimulation and optimal yield. For hyper-responders, preventing ovarian hyperstimulation syndrome (OHSS) is critical; however, for poor responders, choosing a low stimulation protocol aligned with the predicted oocyte yield is considered beneficial. Although there is no universal dosage algorithm because of the involvement of multiple factors, the anti-Müllerian hormone (AMH) level is a key determinant of the appropriate dosage [1-4].

Recombinant follicle-stimulating hormone (rFSH) has been clinically researched since 1985, and it became commercially available in 1996. rFSH has gained widespread use, primarily in Europe and the United States. In Europe, rFSH is the first-line gonadotropin preparation for ovulation induction after cessation of the production and supply of urinary-derived follicle-stimulating hormone (FSH) and human menopausal gonadotropin preparations because of concerns regarding bovine spongiform encephalopathy [5].

Currently, rFSH is the primary agent used for COS with assisted reproductive technology procedures. The first rFSH injection used for COS with in vitro fertilization was follitropin alfa, which is produced using Chinese hamster ovary cells [6]. The newest rFSH injection, follitropin delta (Rekovelle), which is expressed in the human fetal retinal cell line, received marketing authorization in Europe in 2016, and it was introduced in Japan in October 2021.

Rekovelle has characteristics that distinguish it from traditional rFSH preparations, including its human cell-based origin and dose determination based on AMH levels and body weight. According to meta-analyses, both follitropin alfa and follitropin delta demonstrated equivalent pregnancy and live birth rates, and their non-inferiority has been established. Additionally, the incidence of early OHSS with follitropin delta is lower than that with follitropin alfa [7,8].

In this study, we compared the outcomes of follitropin delta and follitropin alfa by performing crossover administration for the same patients. Based on these results, we evaluated the clinical applicability and unique characteristics of these rFSH preparations.

## Materials and methods

Study population

Patients were treated between 2022 and June 2024. This investigation was conducted as a retrospective study. Even though this was a retrospective study, we considered IRB approval to be necessary and therefore submitted the protocol for review by our institutional review board. To protect personal information, all treatment outcomes were reported in a manner that does not allow for individual identification. As the study involved only past clinical data without any medical intervention, it was determined that there would be no associated risks or disadvantages to the participants. This study was approved by the Ethical Committee of Hanabusa Women’s Clinic (ethical review number 2025-4).

Eligible participants were aged 25-42 years, had a BMI of 18-30 kg/m^2^, and underwent COS within four months after the first oocyte retrieval cycle. Patients with AMH levels ≤2.03 ng/mL were divided into four groups based on the crossover use of two rFSH preparations. The minimum AMH level was set at 0.5 ng/mL or higher [9]. At our institution, we initiate controlled ovarian hyperstimulation (COH) even in patients with an AMH level around 0.5 ng/mL. Given the potential for a low oocyte yield in such cases, we presumed COH would be appropriate only when at least four oocytes were likely to be retrieved.

The same patients underwent two treatment cycles. This analysis was conducted on those who did not achieve pregnancy in the first cycle. Patients who received follitropin alfa during both cycles comprised the GG (follitropin alfa administered in both cycles) group (81 patients). Patients who received follitropin delta during both cycles comprised the RR (follitropin delta administered in both cycles) group (51 patients). Patients who received follitropin delta during the first cycle and follitropin alfa during the second cycle comprised the RG (follitropin delta in the first cycle followed by follitropin alfa) group (30 patients). Patients who received follitropin alfa during the first cycle and follitropin delta during the second cycle comprised the GR (follitropin alfa in the first cycle followed by follitropin delta) group (44 patients). Patients who underwent preimplantation genetic testing for aneuploidy were excluded.

AMH measurements

AMH levels were measured using the Elecsys AMH Plus assay (Basel, Switzerland: Roche Diagnostics GmbH) and a Cobas e411 analyzer (Basel, Switzerland: Roche Diagnostics GmbH), both of which employ the electrochemiluminescence immunoassay method.

Stimulation protocol

All patients underwent COS using the progestin-primed ovarian stimulation protocol. Follitropin alfa was administered at a daily dose of 225 IU, whereas follitropin delta was administered at 12 µg per day. Although follitropin alpha and follitropin delta are measured in different units, a study compared these two using the number of oocytes retrieved. In that study, which analyzed data from 1,591 cases, it was concluded that a clinical equivalent of 10 µg of follitropin delta corresponds to 150 IU of follitropin alpha. In our present analysis, we included cases in which 12 µg of follitropin delta (Rekovelle) was used, which is approximately equivalent to 180 IU of follitropin alpha [10]. However, as per clinical practice, the next standard dose above 150 IU is generally 225 IU; we followed this convention in our comparisons.

Starting on day three of the menstrual cycle, patients received daily rFSH injections and 5 mg of medroxyprogesterone acetate (MPA). Follicular development was assessed by transvaginal ultrasound on days 8 and 10, and triggering was initiated when at least two dominant follicles ≥18 mm were observed. MPA was continued until two days before oocyte retrieval. Triggering was primarily achieved using a gonadotropin-releasing hormone antagonist flare protocol with nasal buserelin (buserelin nasal spray 0.15%, 10 mL) administered as two sprays (300 µg each) at 7:00 PM and 8:00 PM on the day of oocyte pick-up. For some patients, 5,000 IU of human chorionic gonadotropin (hCG5000; Tokyo, Japan: Fuji Pharma) or Ovitrelle Pen 250 µg (Rome, Italy: Merck Serono S.p.A.) was used instead of a gonadotropin-releasing hormone agonist. Follicular development was monitored using transvaginal ultrasound, and oocyte retrieval was performed between 8:30 AM and 9:30 AM. All embryos were scheduled to undergo cryopreservation because of the use of MPA.

In vitro fertilization and intracytoplasmic sperm injection procedures

For all groups, the duration of FSH and MPA administration, total FSH dose, and outcomes such as the numbers of retrieved oocytes, degenerated oocytes, fertilized oocytes, and blastocyst formation were analyzed. The intracytoplasmic sperm injection rate was consistently higher than the in vitro fertilization rate across all groups and cycles. Blastocyst formation was assessed on day five or six after retrieval, and blastocyst quality was graded using the Gardner criteria. Blastocysts graded as ≥3 with inner cell mass and trophectoderm scores of at least B or higher were defined as good-quality blastocysts.

A standard deviation of 3.75 for the oocyte count variation of the GG and RR groups was observed. The same rFSH was used for both groups during the first and second trials, and the difference in the number of oocytes retrieved was analyzed. Therefore, a difference of four or more oocytes was considered statistically significant and applied to the results of the GR and RG groups.

Statistical analysis

The statistical analysis was conducted using EZR software (Saitama, Japan: Jichi Medical University Saitama Medical Center). We used the Kruskal-Wallis test, a non-parametric method employed for comparing three or more independent groups. In another part of the analysis, the chi-square test was used. Statistical significance was set at p<0.05.

## Results

Background

The baseline characteristics of the GG, RR, RG, and GR groups are summarized in Table 1. The mean ages of patients in the GG, RR, RG, and GR groups were 37.1, 37.9, 36.6, and 36.0 years, respectively, with no significant differences between groups. Similarly, there were no significant differences in height, weight, infertility causes, menstrual cycle characteristics, and infertility duration. The average antral follicle counts of the GG, RR, RG, and GR groups were 9.8, 10.2, 8.3, and 10.6, respectively, and their average AMH levels were 1.5, 1.4, 1.4, and 1.4 ng/mL, respectively, with no significant differences across groups. Baseline hormone levels (FSH, luteinizing hormone, and testosterone) were also comparable across these groups.

**Table 1:** Demographics and baseline characteristics. GG: follitropin alfa administered in both cycles; RR: follitropin delta administered in both cycles; GR: follitropin alfa in the first cycle followed by follitropin delta; RG: follitropin delta in the first cycle followed by follitropin alfa; FSH: follicle-stimulating hormone; LH: luteinizing hormone

Variables	GG (n=81)	RR (n=51)	RG (n=30)	GR (n=44)
Age (years)	37.1±4.3	37.9±3.0	36.6±3.7	36.0±3.7
<35 years	24	15	6	10
35-37 years	15	10	13	16
38-40 years	20	13	5	15
41 years	22	13	6	3
Height (cm)	158.6±5.7	158.9±7.1	159.4±5.3	157.8±6.4
Weight (kg)	51.9±6.9	52.5±4.7	52.6±7.4	52.6±7.1
Causes of infertility				
Unknown	20	16	6	8
Tubal factor	46	28	16	26
Male factor	7	2	2	4
Endometriosis	4	1	2	4
Others	4	4	4	2
Primary infertility	52	45	24	34
Secondary infertility	29	6	6	10
Duration of infertility (months)	19.6±20.4	24.0±22.4	25.5±18.3	24.3±24.2
AFC	9.8±2.8	10.2±0.4	8.3±3.6	10.6±4.9
AMH (ng/mL)	1.5±0.4	1.4±0.4	1.4±0.3	1.4±0.4
0.5≤1.0	5	7	1	7
1.0≤1.5	31	20	15	15
1.5≤2.03	45	24	14	22
Baseline FSH	6.4±2	5.9±2.4	6.5±3.6	5.9±1.8
Baseline LH	3.8±1.8	4.2±2.0	5.6±4.9	4.9±2.7
Testosterone (T)	20.8±9.6	20.1±6.8	25.1±11.6	22.9±8.9
Menstrual cycle				
Regular	51	41	22	32
Irregular	30	10	8	12

Controlled ovarian stimulation and oocyte retrieval

The duration of FSH administration, total FSH dose, and duration of MPA administration were similar among the GG, RR, RG, and GR groups. The number of retrieved oocytes increased during the second retrieval cycle across all groups (Table 2). The variation in the number of retrieved oocytes between the first and second cycles in the GR group appeared to be higher (1.7 oocytes) compared to other groups (0.8-1.2 oocytes); however, this difference was not statistically significant. The retrieval efficiency, which was calculated as retrieved oocytes/punctured follicles × 100, was not significantly different across groups. The number of degenerated oocytes was also similar across groups. No cases of OHSS or hospitalizations were reported because this study only included patients with AMH levels ≤2.03 ng/mL.

**Table 2:** The rFSH dose, duration of administration, number of oocytes retrieved, and MII rates were presented for each group. *The result of the Kruskal-Wallis test was p=0.831. GG: follitropin alfa administered in both cycles; RR: follitropin delta administered in both cycles; GR: follitropin alfa in the first cycle followed by follitropin delta; RG: follitropin delta in the first cycle followed by follitropin alfa rFSH: recombinant follicle-stimulating hormone; MPA: medroxyprogesterone acetate; OPU: oocyte pick-up

Variables	GG 1st	GG 2nd	RR 1st	RR 2nd	RG 1st	RG 2nd	GR 1st	GR 2nd
rFSH administration days	8.3±1.4	8.5±1.8	8.0±1.5	8.3±1.6	8.7±1.2	8.4±0.9	8.5±1.5	7.8±1.4
MPA administration days	7.7±1	7.7±1	7.1±1.4	7.4±1.4	7.2±2.7	7.7±1	7±1.9	7.8±1.4
rFSH total dosage (G=IU, R=μ)	1923±295	1958±390	100.5±19.3	100.6±7.5	1968±284	101.6±10.9	102±19	2001±491
Number of retrieved oocytes	7.9±3.7	8.3±4.3	6.7±4.4	7.8±4.6	6.3±3.4	7.1±3.1	5.8±3.1	7.5±4.9
Number of degenerated oocytes	0.1±0.3	0.2±0.5	0.1±0.2	0.1±0.3	0.1±0.2	0.1±0.3	0.2±0.5	0.03±0.1
Oocyte retrieval puncture rate	60.5±18.1	66.6±23.1	63.2±20.6	70.1±24.5	57.3±17.2	63.5±19.9	64.1±23.2	68.4±30.1
Number of metaphase II oocytes (MII)	4.9±2.9	5.4±2.9	4.5±3.1	5.1±3.3	4.3±2.5	4.9±2.5	3.4±2.8	5.6±4.4
Difference in number of retrieved oocytes between 1st and 2nd OPU	0.8±3.9*		1.2±3.6*		0.8±3.3*		1.7±3.9*	
MII rate	54.1±33.3	58.3±35.1	59.8±32.8	64.5±24.7	55.0±36.0	62.7±29.3	47.3±31.8	66.8±36.4

Culture outcomes

We collected data regarding MII oocytes, fertilized oocytes, cleaved embryos, blastocysts, and good-quality blastocysts, as well as the differences in the numbers of oocytes retrieved during the first and second retrieval cycles (Table 3). The number of retrieved oocytes, blastocyst formation rate, and good-quality blastocyst formation rate across groups were increased during the second retrieval cycle; however, no significant differences in these rates were observed across each group.

**Table 3:** ART method and culture outcomes (fertilization, cleavage, blastocyst) for each group. GG: follitropin alfa administered in both cycles; RR: follitropin delta administered in both cycles; GR: follitropin alfa in the first cycle followed by follitropin delta; RG: follitropin delta in the first cycle followed by follitropin alfa ART: assisted reproductive technology; ICSI: intracytoplasmic sperm injection; IVF: in vitro fertilization

Variables	GG 1st	GG 2nd	RR 1st	RR 2nd	RG 1st	RG 2nd	GR 1st	GR 2nd
ART method								
ICSI (%)	43 (53.1)	48 (59.3)	36 (70.5)	41 (80.3)	28 (63.6)	35 (79.5)	15 (50.0)	23 (76.7)
IVF (%)	11 (13.6)	13 (16.0)	5 (9.8)	2 (3.9)	10 (22.7)	4 (9.1)	5 (16.7)	2 (6.7)
Split (%)	27 (33.3)	20 (24.7)	10 (19.6)	8 (15.8)	6 (13.6)	5 (11.4)	10 (33.3)	5 (6.7)
No fertilization (%)	1 (1.2)	0 (0.0)	2 (3.9)	2 (3.9)	1 (2.2)	0 (0)	2 (6.7)	1 (3.3)
Number of fertilized eggs	4.7±2.6	5.7±2.8	3.7±2.6	4.3±2.5	3.5±2.2	4.2±2.2	3.4±2.8	5.1±3.3
Number of cleaved embryos	4.3±2.6	5.3±2.7	3.3±2.5	4±2.2	3.5±2.2	3.8±2	3±2.6	4.8±3.2
Number of blastocysts	2.6±2.6	3.1±1.9	1.9±1.6	2.2±1.4	1.9±1.3	2.3±1.4	2±1.7	2.9±2.1
Number of good blastocysts	1.5±1.5	2.2±1.5	1.1±1.1	1.3±1.1	0.8±0.8	1.5±1.1	1.1±1.1	2.2±2.1
Fertilization rate	63.6±25.3	72.3±19.8	60.4±29.4	57.8±24.4	61.6±23.2	59.4±25.6	56.5±29.1	67.1±28.1
Cleavage rate	88.9±18.7	91.3±14.3	90.3±19.8	44.5±12.3	89.7±20.4	88.6±21.5	83.8±28.4	93.5±19.7
Blastocyst formation rate	49.5±30.4	55.1±23.3	44.4±31.8	52±29.9	50±31.6	54.6±33.6	48.2±35	54.8±28.5
Good blastocyst rate	31.4±27.6	40.5±25.2	26.5±28.8	29±24.5	25.3±27	37.3±29.5	29.1±29.7	41.6±31.4

Changes in the retrieved oocyte count

For the GG group, the rate of change in oocyte retrieval was 40.7%, which was the most significant increase between the first and second retrieval cycles (Table 4). For the other groups, the rate of change in oocyte retrieval was approximately 30%; no statistically significant differences were observed across these groups. The percentage of patients with increased oocyte numbers was similar across all groups. The decreased oocyte retrieval rate in the GR group was 4.5%, which was the lowest among all groups and significantly lower than that of the GG group (18.5%).

**Table 4:** Cases with changes in the number of retrieved oocytes (%). A comparison of the oocyte retrieval reduction rates showed no significant difference between the GG and RR groups or between the GG and RG groups, whereas the GR group had a significantly lower reduction rate compared to the GG group (p=0.029). GG: follitropin alfa administered in both cycles; RR: follitropin delta administered in both cycles; GR: follitropin alfa in the first cycle followed by follitropin delta; RG: follitropin delta in the first cycle followed by follitropin alfa

Variables	GG	RR	RG	GR
	n=81	n=51	n=30	n=44
Cases with changes in the number of retrieved oocytes (%)	33 (40.7)	15 (29.4)	10 (33.3)	12 (27.2)
Cases with an increase in the number of retrieved oocytes (%)	18 (22.2)	9 (17.6)	7 (23.3)	10 (22.7)
Cases with a decrease in the number of retrieved oocytes (%)	15 (18.5)	6 (11.1)	3 (10.0)	2.0 (4.5)

## Discussion

Recently, an increasing number of studies have been published comparing follitropin delta and follitropin alpha. In these reports, follitropin alpha is typically administered using a fixed-dose regimen, while follitropin delta is dosed based on an algorithm. The outcomes obtained in both groups have been found to be comparable. Several studies have demonstrated that the algorithm-based dosing of follitropin delta is not inferior to the individualized dosing of follitropin alpha/beta, and it also offers the advantage of objectivity [11-13].

This study aimed to examine the utility of follitropin alfa and follitropin delta in the same patients by altering the type of rFSH used for two oocyte retrieval cycles. While previous studies have evaluated stimulation protocols using different recombinant FSH preparations, few have examined their effects across consecutive cycles in the same individuals using a crossover design [14].

rFSH is produced using Chinese hamster ovary cells, and both follitropin alfa and follitropin beta are derived from these cells. Follitropin alfa is administered in international units (150 IU or 225 IU). Follitropin delta is derived from human embryonic retinoblast cells. In contrast, follitropin delta is unique because its dosage (in μg) is determined using an algorithm based on AMH levels and body weight [2]. Currently, three rFSH preparations, follitropin alfa, follitropin beta, and follitropin delta, are available, and their comparisons have been published [15]. In Japan, studies have suggested that follitropin delta can improve pregnancy rates and reduce the risk of OHSS [15].

In this study, we evaluated oocyte yield variability associated with alternating rFSH preparations (follitropin alfa and follitropin delta) during different cycles in the same patients. Although the dosage units of follitropin alfa and follitropin delta differ, this study approximated equivalence by comparing 225 IU of follitropin alfa with 12 μg of follitropin delta according to the clinical standard of use. Prior reports have suggested that 1 μg of follitropin delta is approximately equivalent to 15 IU of follitropin alfa; therefore, 12 μg is approximately equivalent to 180 IU [10]. Although follitropin delta appeared to be slightly more potent, our results showed no significant increase in oocyte retrieval in the GR group compared to that in the other groups and no significant decrease in the RG group.

In the present study, the design aimed to closely reflect real clinical practice in line with the two rFSH injections. While the fixed-dose regimen for follitropin alpha was consistent with its routine clinical outcome, some studies have reported that follitropin delta offers greater objectivity, even though the clinical outcomes are comparable between the two [11].

A previous study of 49 healthy women aged 21-39 years with endogenous FSH suppression reported that repeated daily administration of 13.5 μg (225 IU) of follitropin delta resulted in a 1.6-fold increase in the peak serum FSH concentration, a 0.6-fold reduction in FSH clearance, and a 2.10-fold increase in the peak estradiol concentration compared to those of follitropin alfa [10]. These differences highlight the need for dose adjustments when clinically applying follitropin delta. The dosing system of follitropin delta is weight-based, whereas that of follitropin alfa is not; therefore, the use of follitropin delta ensures more consistent outcomes.

A dose-response phase II trial involving Japanese women aged 20-39 years compared follitropin delta doses of 6, 9, and 12 μg with a follitropin beta dose of 150 IU and revealed that the number of retrieved oocytes was similar across groups; 12 μg of follitropin delta yielded 11.6±5.6 oocytes, whereas 11 oocytes were yielded in the control group [16]. These results align with those of the present study in which AMH-limited cases were treated with higher doses of follitropin delta (12 μg) or moderately increased doses of follitropin alfa (225 IU).

Phase II and III clinical trials demonstrated dose responsiveness with a follitropin delta dose range of 6-12 μg, thus establishing its efficacy and safety. Furthermore, its non-inferiority to follitropin alfa and follitropin beta was confirmed in terms of clinical pregnancy rates and oocyte retrieval outcomes. Additionally, during previous studies, follitropin delta was associated with a significantly lower incidence of OHSS than that associated with other rFSH preparations [17,18]. However, in those studies, no OHSS cases were observed, likely because of the inclusion of patients with AMH levels ≤2.03 ng/mL.

Although variability in oocyte retrieval was observed with different rFSH preparations and administration sequences, no significant differences in blastocyst formation, good-quality blastocyst rates, or pregnancy rates were observed. These findings suggest that both rFSH preparations can be used interchangeably during assisted reproductive technology procedures without compromising outcomes.

Finally, although the number of retrieved oocytes tended to increase during the second retrieval cycle across all groups, this tendency was greater in the GR group (follitropin alfa during the first cycle and follitropin delta during the second cycle). These findings suggest that using follitropin delta during the second cycle may improve patient outcomes. Further investigations are required to confirm these results.

## Conclusions

In cases where the first in vitro fertilization (IVF) cycle does not result in pregnancy, it is important to evaluate whether the stimulation protocol should be repeated or modified in subsequent cycles. In recent years, recombinant FSH (rFSH) has been used in the majority of stimulation regimens and is considered indispensable. Therefore, we examined whether the type of rFSH used affects the number of oocytes retrieved and the subsequent culture outcomes. Our study concluded that in non-polycystic ovary syndrome (PCOS) patients with an anti-Müllerian hormone (AMH) level of ≤2.03 ng/mL - in intermediate responders - when follitropin delta (12 μg) was used in the second IVF cycle, the rate of change in the number of retrieved oocytes was significantly smaller compared to the first cycle. Follitropin delta may contribute to a more consistent oocyte yield compared to the first cycle.

## References

[1] Barad DH, Weghofer A, Gleicher N (2009). Comparing anti-Müllerian hormone (AMH) and follicle-stimulating hormone (FSH) as predictors of ovarian function. Fertil Steril.

[2] Lee TH, Liu CH, Huang CC (2008). Serum anti-Müllerian hormone and estradiol levels as predictors of ovarian hyperstimulation syndrome in assisted reproduction technology cycles. Hum Reprod.

[3] Broer SL, Broekmans FJ, Laven JS, Fauser BC (2014). Anti-Müllerian hormone: ovarian reserve testing and its potential clinical implications. Hum Reprod Update.

[4] Seifer DB, Baker VL, Leader B (2011). Age-specific serum anti-Müllerian hormone values for 17,120 women presenting to fertility centers within the United States. Fertil Steril.

[5] Lunenfeld B (2012). Gonadotropin stimulation: past, present and future. Reprod Med Biol.

[6] Goa KL, Wagstaff AJ (1998). Follitropin alpha in infertility: a review. BioDrugs.

[7] Qiao J, Zhang Y, Liang X (2021). A randomised controlled trial to clinically validate follitropin delta in its individualised dosing regimen for ovarian stimulation in Asian IVF/ICSI patients. Hum Reprod.

[8] Blockeel C, Griesinger G, Rago R, Larsson P, Sonderegger YL, Rivière S, Laven JS (2022). Prospective multicenter non-interventional real-world study to assess the patterns of use, effectiveness and safety of follitropin delta in routine clinical practice (the PROFILE study). Front Endocrinol (Lausanne).

[9] Revelli A, Biasoni V, Gennarelli G, Canosa S, Dalmasso P, Benedetto C (2016). IVF results in patients with very low serum AMH are significantly affected by chronological age. J Assist Reprod Genet.

[10] Arce JC, Larsson P, García-Velasco JA (2020). Establishing the follitropin delta dose that provides a comparable ovarian response to 150 IU/day follitropin alfa. Reprod Biomed Online.

[11] Gazzo I, Bovis F, Colia D, Sozzi F, Costa M, Anserini P, Massarotti C (2024). Algorithm vs clinical experience: controlled ovarian stimulations with follitropin delta and individualised doses of follitropin alpha/beta. Reprod Fertil.

[12] Haakman O, Liang T, Murray K (2021). In vitro fertilization cycles stimulated with follitropin delta result in similar embryo development and quality when compared with cycles stimulated with follitropin alfa or follitropin beta. F S Rep.

[13] Porcu-Buisson G, Maignien C, Swierkowski-Blanchard N (2024). Prospective multicenter observational real-world study to assess the use, efficacy and safety profile of follitropin delta during IVF/ICSI procedures (DELTA Study). Eur J Obstet Gynecol Reprod Biol.

[14] Vidal MD, Martínez F, Rodríguez I, Polyzos NP (2024). Ovarian response and embryo ploidy following oral micronized progesterone-primed ovarian stimulation versus GnRH antagonist protocol. A prospective study with repeated ovarian stimulation cycles. Hum Reprod.

[15] Palomba S, Caserta D, Levi-Setti PE, Busnelli A (2024). Efficacy and safety of follitropin delta for ovarian stimulation in vitro fertilization/intracytoplasmic sperm injection cycles: a systematic review with meta-analysis. J Ovarian Res.

[16] Olsson H, Sandström R, Grundemar L (2014). Different pharmacokinetic and pharmacodynamic properties of recombinant follicle-stimulating hormone (rFSH) derived from a human cell line compared with rFSH from a non-human cell line. J Clin Pharmacol.

[17] Ishihara O, Klein BM, Arce JC (2021). Randomized, assessor-blind, antimüllerian hormone-stratified, dose-response trial in Japanese in vitro fertilization/intracytoplasmic sperm injection patients undergoing controlled ovarian stimulation with follitropin delta. Fertil Steril.

[18] Andersen AN, Nelson SM, Fauser BC, García-Velasco JA, Klein BM, Arce JC (2017). Individualized versus conventional ovarian stimulation for in vitro fertilization: a multicenter, randomized, controlled, assessor-blinded, phase 3 noninferiority trial. Fertil Steril.

